# Creating a platform for collaborative genomic research

**DOI:** 10.23889/ijpds.v1i1.360

**Published:** 2017-04-19

**Authors:** Mark Smithson, James Frost, Julie Williams, Rebecca Simms, Elisa Majounie, Christine Kitchen, Nandini Badarinarayan, Andrew Moreton, Nicola Denning, Christian Bannister

**Affiliations:** 1 Digital Health Labs Limited; 2 Cardiff University

## Objectives

The objective was to deliver a platform to accelerate collaborative research into the genetics of dementia. The project is part of a larger effort to establish a platform for research incorporating components related to epidemiological data, imaging, wearable technology and tissue banking with initiatives to link these different data sets.

The genomics platform aimed to address a number of challenges encountered by researchers:

- Enabling patient-level linkage between genomic and other datasets
- Selection of study cohorts using genotypic and phenotypic characteristics
- Collaborating with other groups to perform large-scale meta analysis
- Supporting repeatable analysis workflows

## Approach

The genomics platform was developed using a mixture of open source, commercial and bespoke components developed for this project. It is designed to securely handle data and scale to cope with increasing data volumes as well as the quantity and complexity of research undertaken.

It is located in a data centre where researchers can access, explore and analyse the data in a secure environment addressing data security and privacy concerns.

The platform integrates with a supercomputer allowing complex analysis of data to be undertaken easily using novel code or predefined workflows.

The platform was designed and developed through close collaboration with a prominent academic research team.

## Results

We have created a collaborative genomics informatics platform that provides efficient and intuitive selection of study cohorts from multiple data sources, with potentially varying data formats and types, based on both genotypic and phenotypic characteristics. Researchers can combine cohorts definitions using set operations and publish these definitions allowing other researchers to apply them to other datasets.

There is an internet accessible portal which allows researchers to share results of their analysis and support meta analysis of this research. A sophisticated search engine allows these results to be found based on genetic information or annotations. This portal accelerates meta-analysis, an intrinsically collaborative endeavour in genomics research.

There is also close integration with HPC (High Performance Computing) resources for computationally expensive tasks. The platform is underpinned by analytical workflows that not only accelerate and enable reproducible research, but reduce the technical barriers to its use.

The platform enables patient-level linkage with other non-genomics data sources through integration with other research platforms. 

## Conclusion

The developed genomics informatics platform provides a step-change in this type of genetic research, accelerating reproducible collaborative research across multiple disparate organisations and data sources, of varying type and complexity.

